# Brace technology thematic series: the progressive action short brace (PASB)

**DOI:** 10.1186/1748-7161-7-6

**Published:** 2012-02-23

**Authors:** Angelo G Aulisa, Giuseppe Mastantuoni, Marco Laineri, Francesco Falciglia, Marco Giordano, Emanuele Marzetti, Vincenzo Guzzanti

**Affiliations:** 1 Orthopaedic Department, Children's Hospital Bambino Gesù, Institute of Scientific Research, P.zza S. Onofrio 4, 00165 Rome, Italy; 2 POR Protesi Ortopediche Romane, Via C.A. Bertini 24, 00137 Rome, Italy; 3 Department of Orthopaedics and Traumatology, University Hospital "Agostino Gemelli", Catholic University of the Sacred Heart, L.go A. Gemelli 1, 00168 Rome, Italy; 4 University of Cassino, Campus Folcara - Via S. Angelo, 03043 Cassino, (FR), Italy

## Abstract

**Background:**

The Progressive Action Short Brace (PASB) is a custom-made thoraco-lumbar-sacral orthosis (TLSO), devised in 1976 by Dr. Lorenzo Aulisa (Institute of Orthopedics at the Catholic University of the Sacred Heart, Rome, Italy). The PASB was designed to overcome the limits imposed by the trunk anatomy. Indeed, the particular geometry of the brace is able to generate internal forces that modify the elastic reaction of the spine. The PASB is indicated for the conservative treatment of lumbar and thoraco-lumbar scoliosis. The aim of this article is to explain the biomechanic principles of the PASB and the rationale underlying its design. Recently published studies reporting the results of PASB-based treatment of adolescent scoliotic patients are also discussed.

**Description and principles:**

On the coronal plane, the upper margin of the PASB, at the side of the curve concavity, prevents the homolateral bending of the scoliotic curve. The opposite upper margin ends just beneath the apical vertebra. The principle underlying such configuration is that the deflection of the inferior tract of a curved elastic structure, fixed at the bottom end, causes straightening of its upper tract. Therefore, whenever the patient bends towards the convexity of the scoliotic curve, the spine is deflected. On the sagittal plane, the inferior margins of the PASB reach the pelvitrochanteric region, in order to stabilize the brace on the pelvis. The transverse section of the brace above the pelvic grip consists of asymmetrical ellipses. This allows the spine to rotate towards the concave side only, leading to the continuous generation of derotating moments. On the sagittal plane, the brace is contoured so as to reduce the lumbar lordosis. The PASB, by allowing only those movements counteracting the progression of the curve, is able to produce corrective forces that are not dissipated. Therefore, the brace is based on the principle that a constrained spine dynamics can achieve the correction of a curve by inverting the abnormal load distribution during skeletal growth.

**Results:**

Since its introduction in 1976, several studies have been published supporting the validity of the biomechanical principles to which the brace is inspired. In this article, we present the outcome of a case series comprising 110 patients with lumbar and thoraco-lumbar curves treated with PASB brace. Antero-posterior radiographs were used to estimate the curve magnitude (C_M_) and the torsion of the apical vertebra (T_A_) at 5 time points: beginning of treatment (t_1_), one year after the beginning of treatment (t_2_), intermediate time between t_1 _and t_4 _(t_3_), end of weaning (t_4_), 2-year minimum follow-up from t_4 _(t_5_). The average C_M _value was 29.3°Cobb at t_1 _and 13.0°Cobb at t_5_. T_A _was 15.8° Perdroille at t_1 _and 5.0° Perdriolle at t_5_. These results support the efficacy of the PASB in the management of scoliotic patients with lumbar and thoraco-lumbar curves.

**Conclusion:**

The results obtained in patients treated with the PASB confirm the validity of our original biomechanical approach. The efficacy of the PASB derives not only from its unique biomechanical features but also from the simplicity of its design, construction and management.

## Introduction

The anatomical changes characterizing the scoliotic spine modify the geometry of the system and induce an alteration of constraint reactions, thereby producing a new model of stress load distribution [1]. More specifically, the lateral deviation and the pathological lordosis cause a bending moment acting on the spine, so that compression and traction forces are exerted on the posterior-lateral aspect of the concavity and on the anterior-lateral aspect of the convexity of the curve, respectively. In addition, the rotation of the scoliotic segment, which is fixed at both ends, imparts a torque to the relative mobility of segments included in the curve, determining a concentration of tensions in certain areas of vertebrae, discs and capsular ligament apparatus, leading to a permanent condition of unstable equilibrium [2].

During the growth, these abnormally distributed forces can produce an asymmetrical development of vertebral bodies and of the neural arch. It follows that the evolution of scoliosis during the growth is the expression of a progressive deformation of the vertebrae included in the curve [1,3-8]. The degree of progression should be considered in relation to the entity of the curve and the intensity of the acting loads. Bearing these considerations in mind, it is clear that the deformations of the scoliotic spine can be modified by mechanical factors independent of etiological mechanisms. The prognostic and therapeutic validity of such assumption has always been accepted and represents the rationale of brace biomechanics.

The orthosis acts on the mechanical behavior of a scoliotic spine by modifying its natural dynamics through external constraints. In addition, the orthesis, by interacting with the trunk, promotes the generation of corrective forces at the level of pads. The mechanical action of a brace must follow a twofold purpose: stabilize the spine during the progression of scoliosis and transmit forces aimed at restoring a normal spinal configuration. These aims are pursued through a dual action:

1) Passive mechanisms produced by the brace/torso interface:

-The stabilization is achieved by constraining the dynamics of the spine and eliminating the load stress concentration in discrete areas through the reduction of loads acting on the spine and the redistribution of residual loads.

-The corrective action is accomplished through forces generated during the brace/torso interaction at the level of pads. The effectiveness of such forces depends on the pad thickness and position as well as on the strap tightening (9,10).

2) Active mechanisms producing internal corrective forces by shifting the trunk away from pressure areas as well as through derotative and lateral deflective movements along the coronal and spinal planes allowed by the brace geometry.

An effective mechanical action promotes vertebral remodeling and the restoration of symmetric vertebral growth, which are essential prerequisites for proper spinal growth and for avoiding the progressive degeneration of the spine [1,6,7]. However, the analysis of the mechanical action of commonly used orthoses shows that the application of corrective forces presents some limitations.

Understanding the biomechanical action of a brace is of particular importance. The transmission of forces requires the identification of suitable gripping points, or constraints, without which traction, deflection and derotation thrusts on the spine would not be applicable. Moreover, the effectiveness of such forces is related to the type of anatomical structures interposed between thrust areas and the spine.

In spite of numerous studies describing bracing biomechanics, no principle, except for the three-point system, seems to be universally accepted. Orthoses based on this principle, although obtaining a substantial stabilization of the curve, are unlikely to correct the deformity. Indeed, the application of external forces, to the extent allowed by existing orthoses, can overcome the phenomenon of concentration of tensions, but does not produce the reversal of stress loads necessary to modify the growth pattern of the scoliotic vertebrae and promote the recovery of the deformity [9,10]. This is due to two factors that limit the efficacy of external forces. The first is of anatomical nature. Braces cannot transfer forces directly onto the spine, but only through the mediation of interposed tissues and organs. Their efficacy is therefore subject to constraints and pressure points, which not always allow the effective transmission of forces. More specifically, the mechanical action is more effective when thrusts are applied to the thoracic cage than on the abdominal cavity. This is linked to the rigidity of the thoracic cage, which reduces the dissipation of applied forces. The second factor concerns the current model of application of external forces through the use of pads, which, because of the visco-elastic response of the spine and the thoracic structures, rapidly deplete their action. This limit is due to the relaxation phenomenon, which occurs when a load is applied to a visco-elastic structure: external forces are dissipated both for shearing and relaxation [11].

In addition, factors related to the cultural background of the orthopedic surgeon may represent a further limitation affecting the efficacy of orthoses. These factors include the clinical experience of the physician and the biomechanical model adopted for the interpretation of the deformity progression. In a SOSORT consensus paper on TLSO biomechanics, Rigo et al. [12] highlighted that a major limitation to the achievement of a "consensus treatment" resides in the diversity of ideas and personal interpretations about the biomechanics of correction, brace design and treatment protocols. These observations emphasize the importance of a better understanding of the biomechanical principles of bracing and underline the need for orthoses able to interfere with the elastic behavior of the deformed spine. Indeed, when the limits imposed by the anatomy are not objectively surmountable, an appropriate brace geometry that constraints the movements of the trunk to exploit the elastic reaction of the system, appears to be the only approach to design braces based on suitable biomechanical principles. The Progressive Action Short Brace (PASB) is indeed inspired to this proposition.

### History

**Figure 1:**
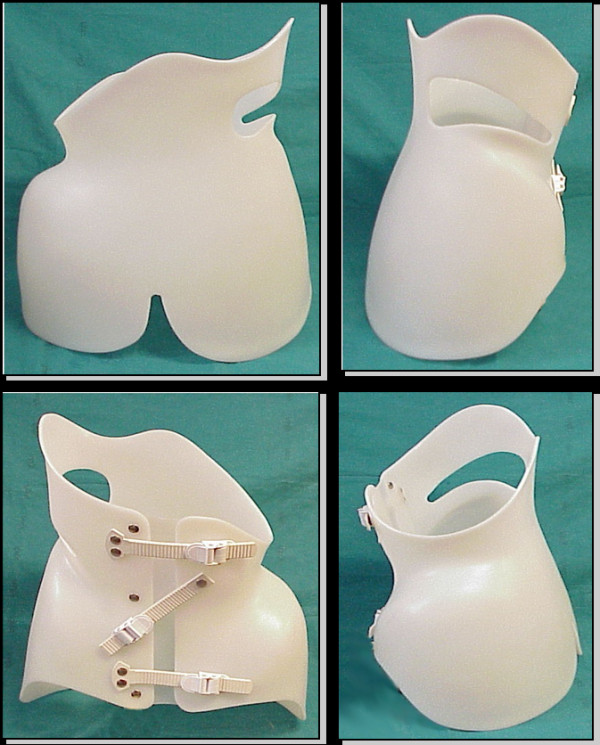
**The Progressive Action Short Brace (PASB)**.

The PASB is a custom-made thoraco-lumbar-sacral orthosis (TLSO), devised in 1976 by Dr. Lorenzo Aulisa at the Institute of Orthopedics, Catholic University of the Sacred Heart of Rome (Figure 1). The PASB was designed to overcome the limits imposed by the anatomy of the trunk for the treatment of lumbar and thoraco-lumbar curves. Indeed, the abdominal cavity reduces the efficiency of forces generated by the brace/torso interaction. The basic principle underlying the rationale of our orthosis resides in its geometry which is able to generate internal forces, such to modify the elastic reaction of the system.

The design of the brace was further improved thanks to the biomechanical studies performed in collaboration with Drs. Di Benedetto and Vinciguerra of the Faculty of Engineering at the "La Sapienza" University of Rome.

The biomechanical principles to which the brace is inspired have been presented at numerous national and international conferences [2,13,14]. Since its introduction, over five-hundred scoliotic patients have been treated in our Department. The efficacy of the PASB has been confirmed both in the short and the long term. Results from clinical trials adopting the PASB have been published in several scientific journals since 1981 [9,15-18].

### Theoretical principles

The brace is based on the biomechanical principle that a constrained spine dynamics can achieve the correction of a curve by inverting the abnormal load distribution during growth.We have hypothesized that another type of forces, namely the internal forces generated by the spine dynamics, can be advantageously used for correction purposes. These forces are not dissipated, as they are constantly generated during the patient's natural movements [13,15,16]. Therefore, the PASB expresses an original biomechanical notion according to which the application of external forces aimed at producing a partial reduction of the deformity is followed by a second phase based on the generation of continuous internal corrective forces.

**Figure 2:**
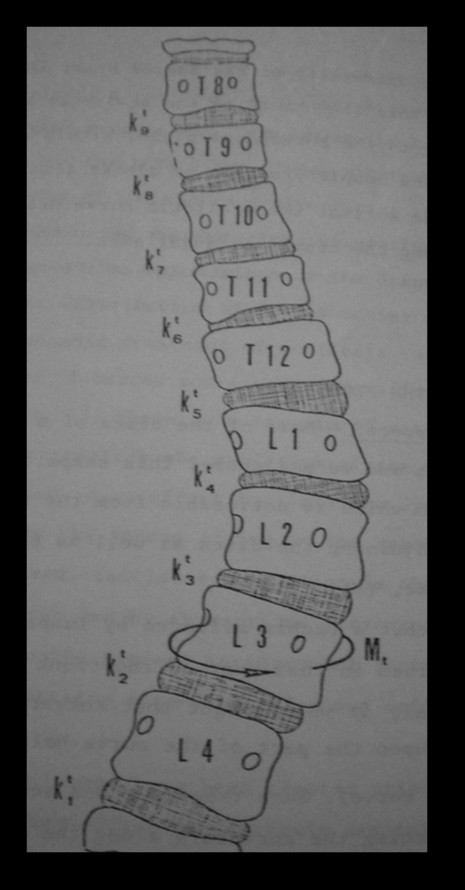
**Rotation of vertebral bodies and pedicle displacements in a case of thoracic-lumbar scoliosis**. The derotation moment (Mt) is applied under the apical vertebra and leads to a derotation of the disks in the upper part of the curve.

This objective is pursued in two phases. The first is finalized to the reduction of the deformity to the extent allowed by the residual curve elasticity through externally applied forces. This rationale of this phase resides in the theory of elasticity, according to which a deformed elastic structure, bound to one extreme and subjected at its bottom to a bending moment and a torque of opposite direction to those that generated the deformation, tends, for elastic reaction, to straighten also in its upper portion. This is what occurs in a scoliotic spine, in which each disc develops on a wedge-shaped and a bent configuration, due to a state of torsion evidenced by the relative rotation of vertebrae adjacent to the disc (Figure 2). Such principle is applicable to thoraco-lumbar and lumbar scoliosis by virtue of the constraint represented by the spine-pelvic junction. Moreover, since an imbalance of the spine at level of the higher loads area contributes to the progression of deformity, it is conceivable the rearrangement of the inferior portion of the curve could help in correction of the whole deformity. These considerations represent the basis of the first phase of the intervention, which ends with the plaster cast manufacturing.

The second phase is based on the principle that internal forces that exert continuous corrective actions are generated by imposing appropriate constraints on the natural dynamics of the spine, allowing the movements only in the direction opposite to the deformity. The dynamic effect of the brace is realized by imposing to the patient's trunk forced directions during daily activities. The natural movement of the trunk towards forced directions produces deflecting and derotating moments that, being generated by a properly bound dynamic, adapt to changes of the system and maintain their efficacy over time. Such principle is reflected in the manufacturing of a plastic brace, made from a plaster cast mould, whose geometry is defined both by surface profiles with adequate edges and by appropriate horizontal sections that promote the development of internal forces by linking the natural spine dynamics [14-16].

**Figure 3:**
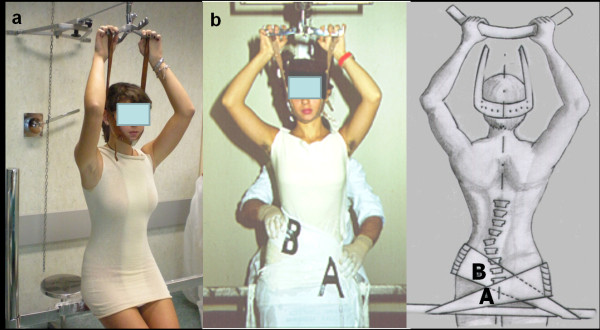
**Brace or plaster cast custom-made**. (a) The patient, in light traction, is positioned with hip and knees slightly bent, in order to obtain the correction of the hyperlordosis. (b) Lateral deflection is achieved by applying a plaster band just beneath the apical vertebra (B). A second band stabilizes the pelvis (A).

The practical application of biomechanical principles of the PASB is achieved through two operative phases. A plaster cast phase precedes the brace application. At this stage, external forces are imparted to correct the flexible component of the deformity. For the manufacturing of the plaster cast, the patient sits on a crossbar, in a gently chin traction, with hips and knees slightly bent, in order to achieve the smoothing of lumbar lordosis (Figure 3a). Subsequently, two plastered bands are applied, attached to the support bar. These bands serve to stabilize the pelvis (plaster band A) and deflect the curve, bringing the vertebrae under the apical vertebra near the cephalo-caudal axis (plaster band B) (Figure 3b).

Afterwards, we proceed to the plaster cast manufacturing, taking care to exert a torque moment of opposite direction to the spinal twist and shaping the hip at the convex side of the curve. Once the plaster cast is completed, before its consolidation, the last thrusts are applied manually.

**Figure 4:**
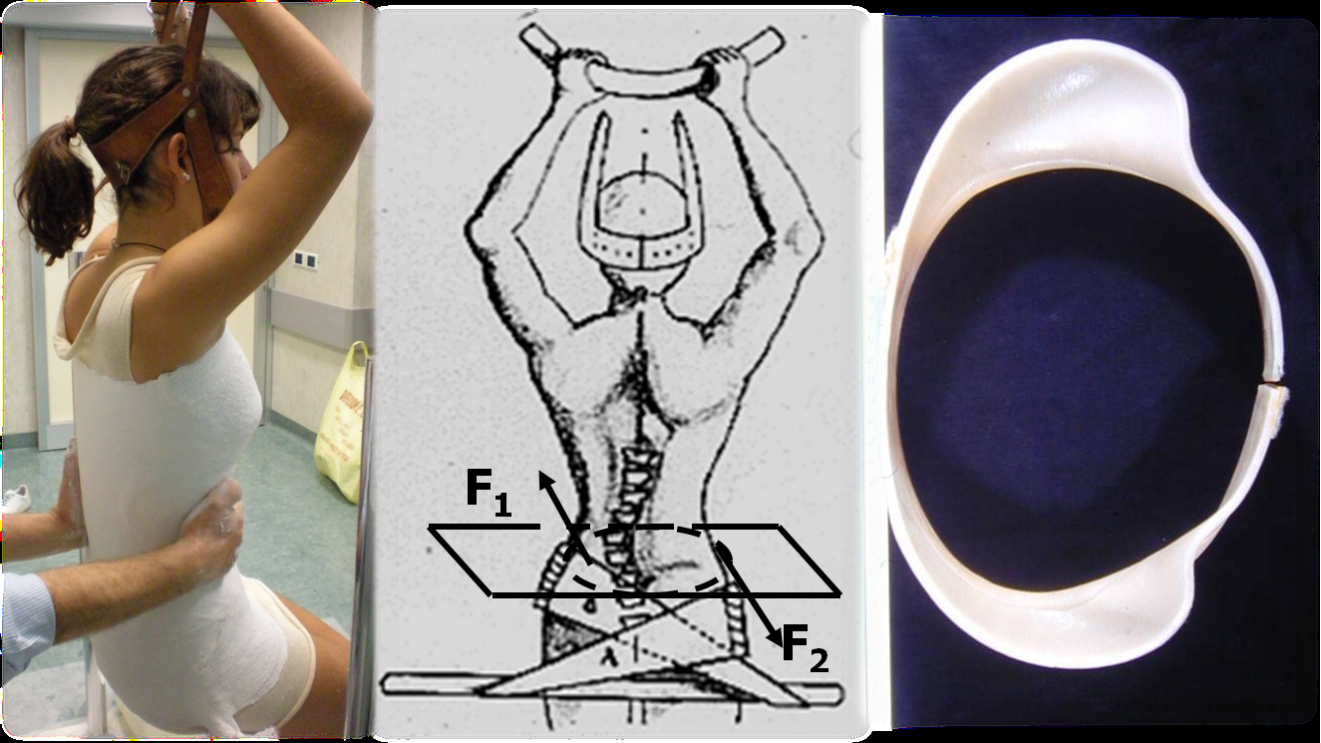
**After the plaster cast is completed, the operator applies a twisting moment**. The direction of the rotation produced by the paired forces is opposite to the direction of the vertebral torsion of the scoliotic curve. This allows to obtain transverse sections represented by asymmetrical ellipses.

**Figure 5:**
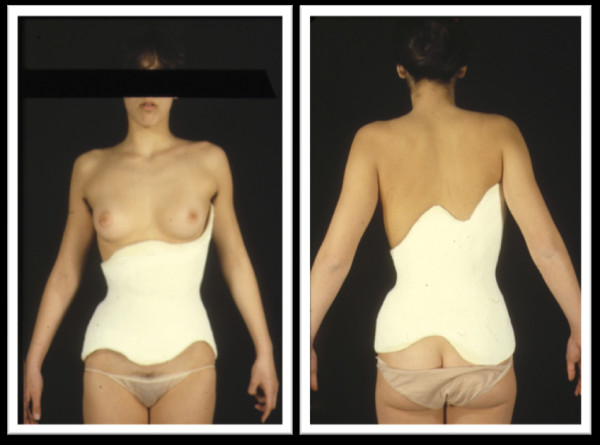
**The finishing touches of the plaster cast establish the same geometry of the plastic brace**.

On the convex side, which corresponds to the spine segment under the curve apex, thrust are directed from the top to the bottom and in a posterior-lateral direction. On the opposite side, a counterthrust is exerted at a higher level, correspondent to the fluctuating ribs. This generates a torque that completes the derotating action of the plaster bands and allows to obtain a consolidated asymmetrical horizontal sections of elliptic shape, necessary for achieving the dynamic correction of the second phase (Figure 4). The finishing touch of the cast establishes the definite geometry of the plastic brace (Figure 5). Sometimes one or two plaster casts, in relation to the curve stiffness, are crafted before switching to the polypropylene orthosis.

**Figure 6:**
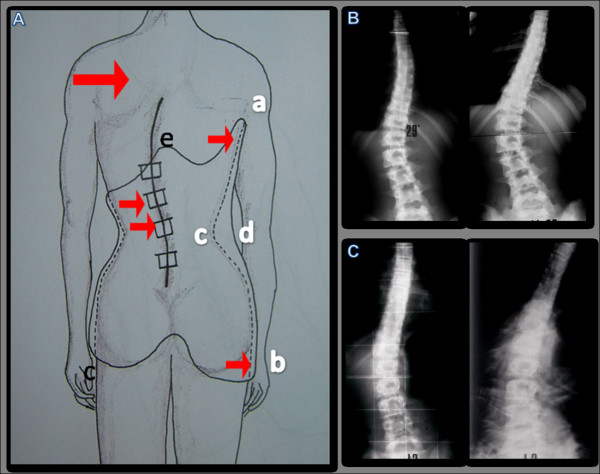
**Dynamics constrained by the brace geometry**. From the concave side (A), the brace extends from the throcanteric region (a) up to the superior neutral vertebra (b). Therefore, the flexion toward the deformity is opposed. The presence of a free space (c-d) between the iliac crest and the upper vertebral limit favors the spine realignment along the cephalo-caudal axis plane (A). The shape of the concave side without (B) and with the brace (C) shows a remarkable difference between the dynamics of the free and that of the constrained spine.

**Figure 7:**
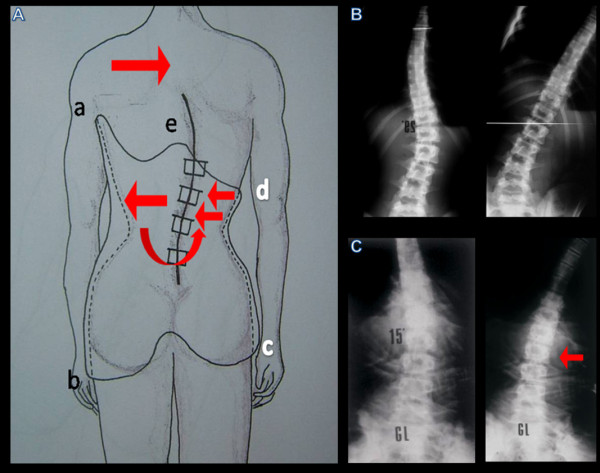
**Dynamics constrained by the brace geometry**. On the convex side, the superior margin ends under the apex vertebra, allowing the lateral flexion movement to induce the deflection of the curve and the realignment of the spine under the apex vertebra. The posterior edge is modeled with a central active prominence which is uncomfortable and compels the patient to perform an anterior translation that allows the hyperlordosis correction (A). Such a profile imposes a particular dynamics, for which the anterior flection movements can be performed only together with a lateral flexion and a rotation in the direction of the correction of the curve. The bending X-ray exam at the convexity side without (B) and with the brace (C) confirms the theoretical assumptions, showing a remarkable difference between the dynamics of the free and the constrained spine.

In the second phase, a plaster mould cast is used for the custom-made PASB manufacturing. The brace mode of action depends on its peculiar geometry, that is determined by the outlines of free ends and by a redistribution of volumes. On the coronal plane (Figure 6), the pelvic grip on the concavity side, extends from the upper trochanteric region to the area above the upper neutral vertebra. By this way, the lateral bending in the direction of the deformity progression is opposed. On the convex side, the free upper edge ends just under the apical vertebra. Such geometry allows the patient to perform lateral flexion movements of the trunk in the direction of the convexity. Due to the constraints imposed by the brace, a dual action is exerted on the scoliotic spine (Figure 7):

1. the deflection of the curve segment above the apical vertebra;

2. the displacement of the inferior tract towards the cephalo-caudal axis.

In the latter tract, the constraint represented by the upper portion of the brace generates a set of forces whose resultant stimulates the recovery of the alignment along the cephalo-caudal axis. The presence of a free volume between the iliac crest and the upper limit vertebra promotes the realignment of the spine along the cephalo-caudal axis. The anterior edge is modeled according to an oblique line, which covers the ribs of the concave side, leaving the contralateral ribs free. Such asymmetrical profile imposes particular dynamics, for which the anterior flexion can only be achieved in combination with a lateral flexion and rotation in the direction of the curve correction.

**Figure 8:**
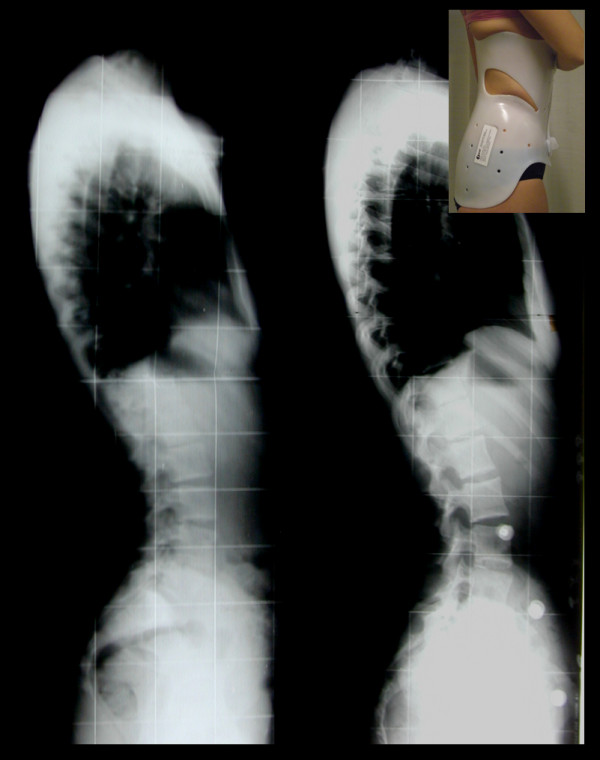
**On the sagittal plane the posterior edge presents a median projection, in order to maintain the flattening of the lumbar lordosis**.

**Figure 9:**
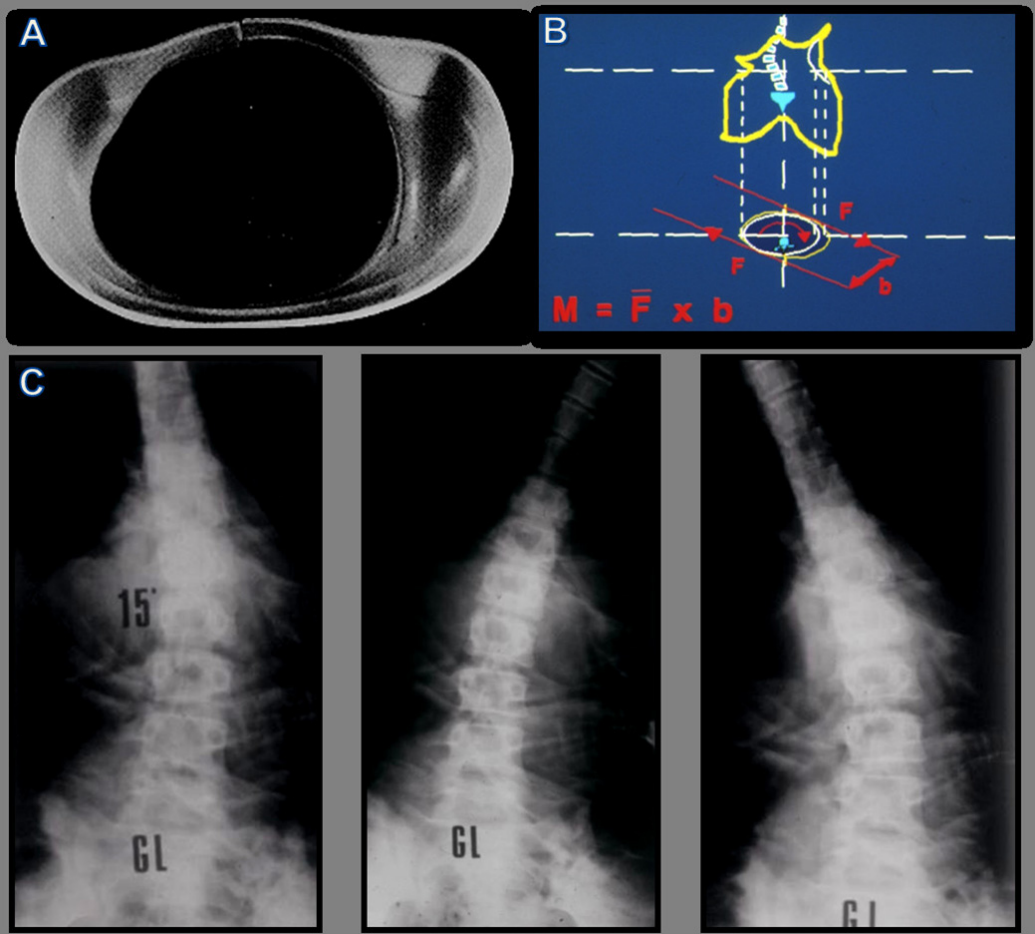
**Dynamics constrained by the brace geometry**. The trasverse section of the brace (A), elliptical and asymmetrical up to the plan of the pelvic hold, produces twisting moments opposed to the direction of rotation of the vertebrae included in the curve (b). The bending X-ray (c) shows the efficacy of the twisting action produced by the brace in all movements of the trunk.

On the sagittal plane (Figure 8), the posterior edge presents a median projection, in order to maintain the flattening of lordosis. The geometry of horizontal sections of the brace is elliptical and asymmetrical above the plane passing through the pelvic grip (Figure 9). This generates derotating moments of opposite direction to the vertebral twist included within the curve.

The closure of the brace is located on the front side and is obtained by three straps. This allows to maintain the proper location of the pads and their intensity, different to what occurs with the posterior closure.

### Practical issues

#### Criteria for PASB bracing and how to prescribe it

In order to determine it the PASB is indicated for the treatment of lumbar and thoraco-lumbar scoliosis, the following radiological parameters are taken into account:

-curve severity, expressed in Cobb degrees;

-curve progression, as indicated by clinical and radiographic examinations to be performed twice a year (5°Cobb change compared with the previous X-ray in a curve over 20°).

-degree of rotation of the apical vertebra, measured in Perdriolle degrees;

-presence of signs of segmental instability.

Once the integrated analysis of these parameters has given the indication for treatment, the patient enters in a treatment protocol comprising two consecutive steps. The first consists in the manufacturing of one or more plaster casts, as described in the relative paragraph, renewed at intervals of two weeks. During this period, one tries to obtain the partial reduction of the deformity, through derotation and deflection actions on the bottom portion of the curve. The renewal of the plaster cast every two weeks is dictated by the rapid depletion of the corrective action of the plaster, because of the visco-elastic response of the system to the stress loads imposed [9]. The indication for treatment with the plaster cast is given by the degree of structuring of the curve referred to the entity of rotation that must be greater than 15° Perdriolle.

**Figure 10:**
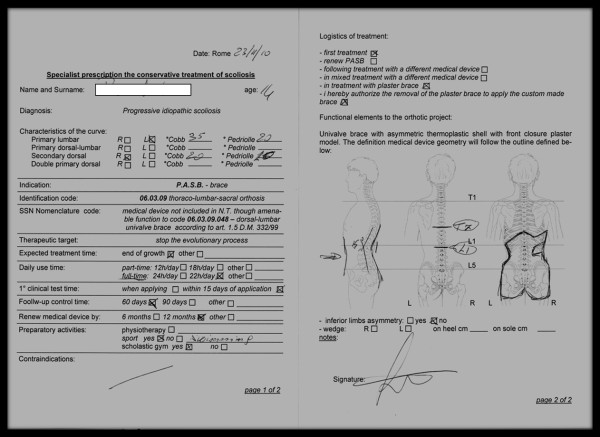
**Card for brace prescription**.

The second step includes the application of the plastic brace, obtained by the plaster cast. For the brace prescription, the following parameters need to be recorded: the side of the curve, the limit vertebra and the apical vertebra. For this purpose, a special card for prescription is used (Figure 10).

#### Contraindications

The PASB brace is not indicated for thoracic or double major scoliosis.

#### Principles of construction of the PASB

The first preliminary step consists in reading the specialist prescription reporting all the information necessary to tailor the brace geometry to the patient's needs (curve type; entity and degree of stiffness; curve's apex vertebra; vertebra apex offset; pelvis anteversion; presence of pelvic dysmorphisms; lower limb dysmetria; duration of brace wearing; timing of clinical controls; first treatment, deriving from other treatment, renewal).

The second step consists in the clinical and radiographic control, followed by the collection of morphometric and lifestyle parameters (e.g., circumference of the trunk, weight, height, activity level) and an interview, which serves to establish an empathic relationship between the specialist and the patient's family and to identify potential factors impacting the compliance to treatment.

#### The plaster model

The method of choice to craft the mould plaster cast of PASB is based on plaster bands (20 cm × 3 m). This approach, contrary to digital technology, allows the direct perception of applied forces by the operator, resulting in an optimal balance between action and derived compensation. In fact, the physician can increase the thrust underneath the apex vertebra until perceiving the depletion of the spinal elastic response. This marks the beginning of a visco-elastic reaction, according to which an increment of the manual force is not accompanied by a proportional increase of curve correction. Hence, the operator can perceive the degree of curve structuring and modulate the forces also as a function of the patient's tolerance.

**Figure 11:**
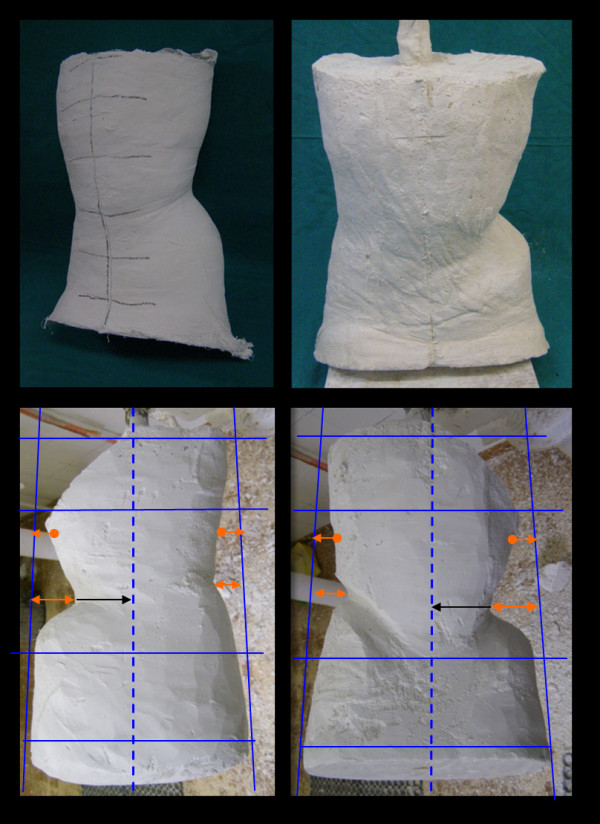
**Plaster model processing phases**.

During the subsequent processing phases of the model, pressure, thrust and expansion areas are further improved in relation to support/stabilization areas. The mould plaster cast (negative model) is closed and isolated in its inner surface to accommodate the plaster mixture necessary for the conformation of the positive model. Once the plaster mixture is solidified, the model is freed and separated from the shell, obtaining a trunk silhouette to work on (Figure 11). The positive model is then compared with measurements obtained during the patient examination and any eventual discrepancies is corrected. The proper cephalo-caudal alignment of the positive is checked both on the coronal and the sagittal plane. On the sagittal plane, a slight paraphysiological lordosis is maintained, variable from case to case, with an average of 20°. On the transverse plane, the model maintains an ovalar section necessary to correct the rotation of the scoliotic spine.

#### Correction/expansion surfaces

**Figure 12:**
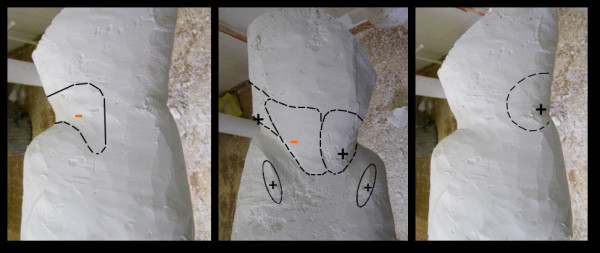
**Correction and expansion surfaces**.

Great attention is paid to the area where the lumbar thrust is applied, which is responsible for the passive correction component (Figure 12). Anteriorly, in the opposite area, the volumetric expansion resulting from the model is enlarged to promote the spine straightening along the cephalo-caudal axis. The expansion chamber on the concave side of the curve towards the posterior-lateral direction is fenestrated to increase the amount of free space needed to straighten the curve.

#### Areas of counterthrust/stabilization

**Figure 13:**
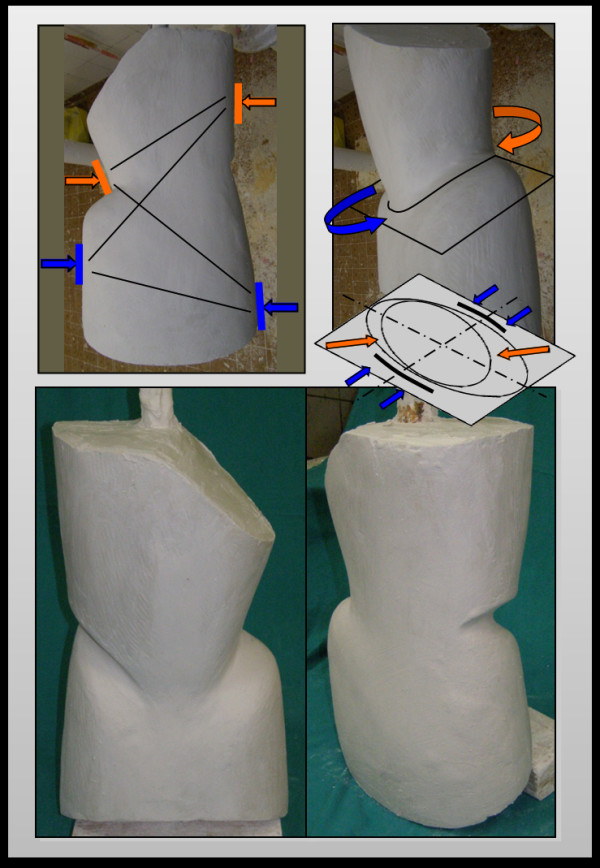
**Areas of counter-thrust and stabilization**.

The PASB, in its unique asymmetric geometry, expresses a large amount of forces and moments which must be balanced with each other by equivalent forces and moments induced by the geometry of the thermoplastic model based on which the PASB is manufactured (Figure 13). On the coronal plane, the pressure area applied to the convexity of the curve on its latero-lateral component is balanced by dorsal, sub-trochanteric and mid-gluteus counterthrusts, and is stabilized by the support supra-trochanteric ipsilateral area. On the sagittal and transverse planes, the posterior-anterior pressure area is balanced by the abdominal and costal counterthrust and is stabilized by the sacro-gluteal support.

In order to obtain an effective abdominal constraint, a substantial trimming of material from this area of the model is necessary to produce a slight undercut (when compatible with the patient's morphology) in relation to the spina iliaca anterior superior (SIAS). A depression beginning at the xiphoid apophysis and ending at the pubic symphysis, limited by the two SIAS, generates a corresponding oval section that avoids the device rotation.

#### Lamination

**Figure 14:**
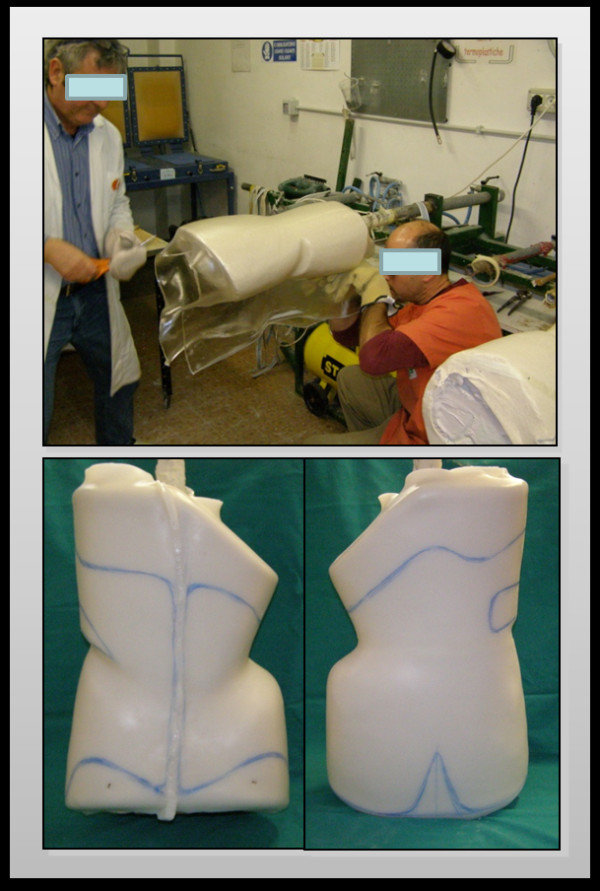
**Lamination and shell cutting**.

The construction of the PASB's shell is made in thermoplastic without internal soft covering. The raw material of choice is laminate (polyolefin) of linear low density polyethylene (food standard), with a thickness of 4 mm. This material presents several advantages, including remarkable lightness, lack of contact toxicity, resistance and good elasticity, faithful copy of the positive model, possibility of small changes of the shell and edges, easy maintenance and inexpensive processing. The thermoforming technique is commonly used for heating in air-oven (about 130°C) and for the following shaping of the model (Figure 14).

Once conformed, the thermoplastic is left to dry for 24 hours on the positive before being removed and sent to the cutting phase in order to limit the effect of the linear shrinkage, typical of linear polyethylene, and loosen internal tensions that may appear during cooling.

#### Shell cutting

Once the manufacture is removed from the positive, a first cut of the shell is made following the profile of the prescribed geometry and defined using the specifics of the case in exam.

#### Static and dynamic tests

The test on the patient is preceded by the identification of cutaneous levels and thrust areas, using a dermographic pencil. Subsequently, the brace is applied to the patient by means of closures temporarily made with adhesive tape. On the frontal plane, the closure tension must ensure the coherent and continuous adhesion of the shell to the skin as well as the support on both trochanters, while imparting the desired position to the lumbar spine. The efficacy of the pressure applied on the lumbar area and the escape towards the expansion areas must be verified. Anteriorly, the finishing and cut line is marked at the bottom to follow the progression of the inguinal fold with the hip flexed and the thigh conformation to the level of the pubic symphysis, while laterally degrades until completely containing the trochanter.

At the concave side, the bottom edge is subtrochanteric and asymmetric relative to the counter-lateral. This portion of the brace is more pronounced to balance the chest counterthrust.

The upper lateral edge at the curve convexity is situated at the level of the apex vertebra and is dulled for about 2 cm, to limit the concentration of thrusts which may cause skin abrasions.

**Figure 15:**
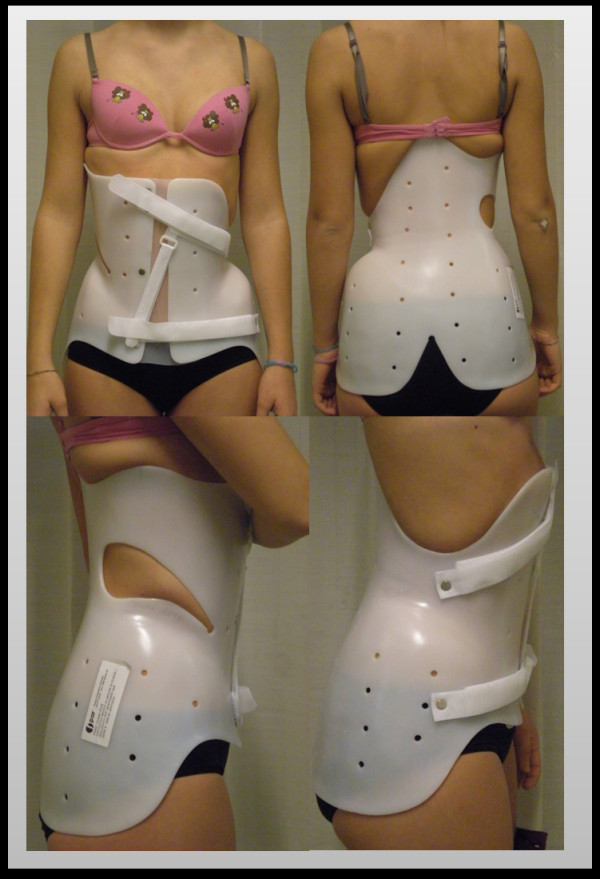
**The brace worn by the patient at the end of tests**.

Posteriorly, the device extends to fully cover the gluteal region. This serves to avoid cosmetic damage (double gluteus, stretch marks, cellulite) and better distribute the pressure resulting from the abdominal thrust and the counter-rotation moment of the lumbar thrust. The gluteus support is marked by a deep central groove to allow a better fit of clothes. The extension of the upper and central prominence, together with the gluteus support, allows the reduction of the pelvis anteversion. It is also important to verify if the posterior edge interferes with sitting, to avoid the displacement of the device in the sitting position. The adhesive tape is finally removed and substituted with velcro closures (Figure 15).

#### Training for the use and maintenance of the PASB

Once completed, the PASB brace is delivered to the patient and a short training on how to wearing it and checking its correct position is offered. The patients is also instructed on how to remove the brace and provide to its cleaning and maintenance. In addition, the patient and his/her family are informed about the need for periodical checks of the brace by the specialist and the possibility that the device may be modified depending on the progression of the scoliotic curve.

#### Protocols (treatment methods)

The term "treatment methods" refers to factors related with timing and modalities of the orthotic treatment from its beginning until completion.

These factors include:

1) The total duration of treatment. This period is further divided into two time intervals:

-from treatment start to the beginning of weaning

-weaning phase and definitive orthosis abandonment.

2) Full-time or part-time brace treatment during the period included between the start of treatment--and the beginning of weaning.

3) Clinical management of the patient during the brace treatment.

#### Total duration of treatment

All authors agree that the orthotic treatment should be continued for the entire period of skeletal growth. In fact, to achieve the remodeling of the segments of movement, the mechanical action of the brace should act as long as vertebral growth cartilages are active. This ensures a certain uniformity regarding the time of completion of the therapeutic program. It should however be emphasized that the skeletal maturation stage is determined by means of indirect indicators, such as the Risser sign and/or the vertebral ring-apophyses ossification, whose correlation, with both the chronological age and the end of spinal growth, presents high interindividual variability. Because of the great variability of the relationship between Risser 4/5 and the patient's age [17-19], we prefer to utilize the fusion of the vertebral ring-apophyses as a parameter to determine the end of spinal growth. Therefore, our patients usually begin the weaning 2-3 years later than those evaluated on the base of a Risser 4 sign [20,21]. This difference is reduced or null in the case of a Risser 5 sign.

#### Weaning phase

The phase of weaning is carried out in different ways by various authors, even if the common denominator is the progressive abandonment of the orthosis. We apply a reduction of multiples of two hours every three months, up to 12 hours out of the brace a day. Subsequently, the orthosis is worn for 6 months during nighttime. The weaning can be temporarily suspended in case of evident progression of the curve.

Most authors requests an X-ray examination of the curve every six months, before increasing the hours of freedom. Such control is usually performed after the patient has been brace-free for at least 4 hours [22]. To reduce the number of X-rays taken, we rely on the hump changes, considering that to a stable measurement of the hump usually corresponds a stable curve [23].

#### Full-time or part-time brace treatments

A full-time orthotic treatment requires that the patient wears the brace for 24 hours a day. In the case of part-time treatment, the patient wears the orthosis for a certain numbers of hours daily, variable for different authors. We use the full-time treatment, because it is the only one that allows us to vary the stress-load distribution in a stable manner and thus to achieve changes of vertebral geometry necessary for the recovery of the curve. In practice, however, the patient has two hours of freedom for his/her personal toilet and to perform physical exercise. Our patients are also allowed to remove the brace for longer periods of time during holidays or special occasions.

During treatment, in case of an evident and stable recovery of the vertebral geometry, we gradually increase the hours of freedom, informing the patient that a return to full-time treatment may be necessary in case the correction is lost.

### Principles of checking

#### Clinical management of treatment

The relaxation phenomenon, which occurs when a load is applied to a visco-elastic structure, causes a progressive reduction of the corrective actions exerted by the brace and requires the continuous restoration of pads [11]. In addition, the somatic growth modifies the relationship between the patient's trunk and the brace. Therefore, it is necessary that the brace efficacy is checked regularly. The frequency of clinical examinations depends on the patient's rate of growth and the structuring degree of the curve. In patients in pre-pubertal age and during the first year post-pubertal, a clinical examination is expected once every two months. The same interval is applied to patients with curves that remain over 30°Cobb and over 15° Perdriolle. For all others, a clinical examination is performed every three months.

Clinical checkups are made by the physician in the presence of an orthopedic technician. Eventual modifications to the brace are made during the visit and verified by the physician. Assessing the efficacy of PASB is extremely simple. Two aspects are to be taken into account:

-the growth of the hip causes a rising of the brace, so that the upper lateral edge, on the convex side, rises above the apical vertebra level.

-the correction of the curve, by causing a realignment of the spine along the cephalo-caudal axis, makes the thrust exerted on the bottom of the convexity less effective.

Therefore, during the examination, it must be checked that the top lateral edge is always located just below the apical vertebra and that the lateral thrust is effective. After the modifications are made, the efficiency of the thrust is evaluated by observing the patient after wearing the brace for at least 10 minutes, by observing the degree of skin redness.

### Exercises

Since the PASB exploits the natural trunk dynamics, patients are not required to perform any programmed exercise over the course of treatment.

## Results & case reports

**Figure 16:**
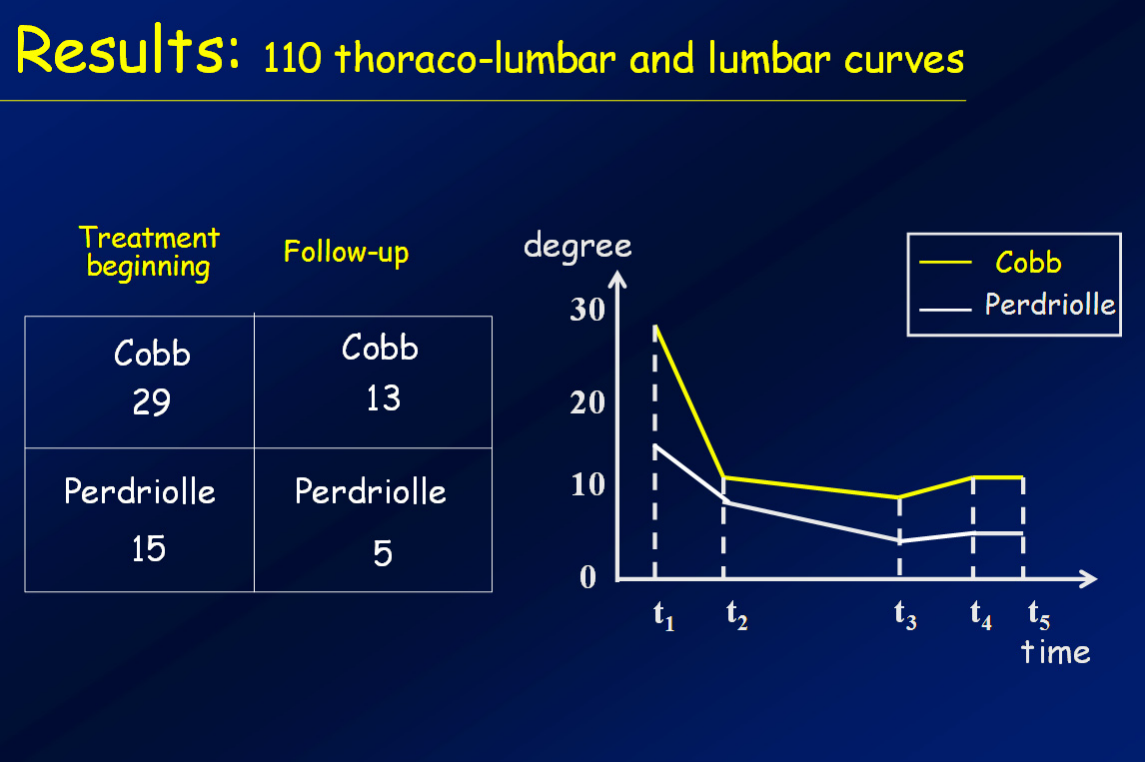
**Results of PASB bracing**.

The results obtained with the PASB have been published in several scientific papers and confirm the validity of the biomechanical principles to which the brace is inspired. Collectively, our results show that the brace is able to interfere with the progression of scoliosis, allowing in many cases a recovery that maintains over time [14,24,25]. In this context, we report the results of a case series comprising 110 consecutive patients with thoraco-lumbar and lumbar curves treated with PASB brace (Figure 16). Data have been extracted from a prospective database. Only patients fully compliant to treatment have been included.

By comparing the first in-brace radiological examination (t_2_) and the X-ray taken 6 months after the beginning of treatment, a remarkable correction is evident both of the lateral deviation, whose average values reduces from 29.3°Cobb to 13.9°Cobb (min 4°, max 23°), and the rotation, that decreases from15.8° Perdriolle to 8.3° Perdriolle (min 3°, max 20°). At the following follow-ups, a further gradual improvement can be observed, especially on derotation. In fact, at the beginning of weaning (t_3_), the following values are observed: 10.6°Cobb (min 3°, max 22°) and 4.5° Perdriolle (min 0°, max 8°). At the end of weaning (t_4_), a moderate loss of correction occurs, mainly as a lateral deviation. In fact, while the lateral deviation increases up to 11.8°Cobb (min 3°, max 24°), with an average loss of 2.4°, the rotation remains substantially unchanged (average 4.9° Perdriolle; min 2°, max 11°). The correction is maintained over a long-term follow-up (t_5_). The lateral deviation settles to an average value of 13°Cobb (min 4°, max 30°), with a mean loss of 1.2°, whereas the rotation stabilizes at 5° Perdriolle, with a non significant average loss of 0.1°.

**Figure 17:**
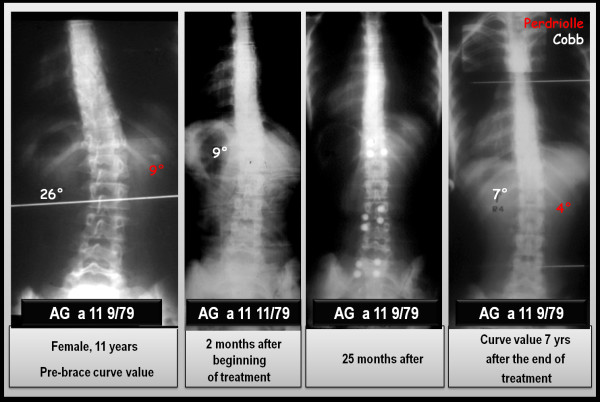
**Typical trend of scoliosis in treatment with the PASB**.

**Figure 18:**
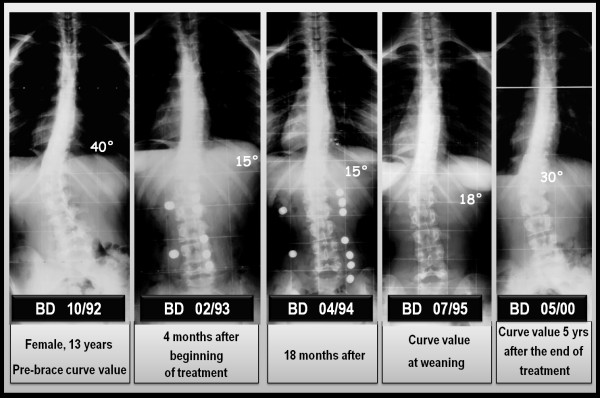
**Example of disc hysteresis with a loss of correction**.

**Figure 19:**
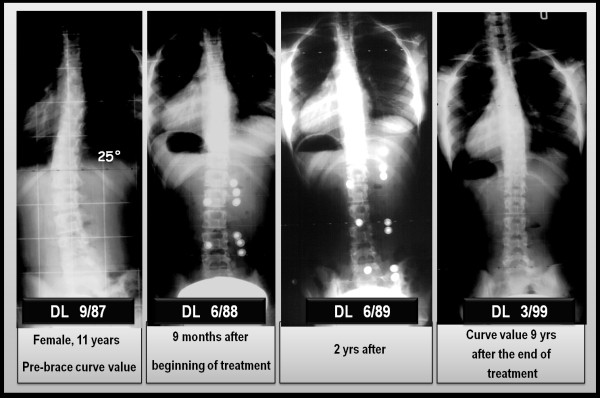
**Example of early weaning in a case of complete recovery of geometry**.

The curve trend, besides confirming the efficacy of PASB bracing, highlights some peculiar features of the brace's action. The reduction of Cobb degrees occurs mostly during the first 6 months of treatment and maintains stable during the following years. The brace action on the rotation is slower, but constant throughout the entire treatment period (Figure 17). Moreover, the correction of rotation is dependent on the initial rotation degree and, therefore, on the residual visco-elastic discal characteristics (Figures 18 and 19). Finally, our data underscore the fact that derotation represents the essential condition for the maintenance of correction [3,26].

### Compliance

The daily hours of bracing (i.e. max 22, min 18) are defined for each patient both in relation to the subject's clinical needs and degree of acceptance. In order to maximize the compliance, patients are always evaluated by the same physician. The presence of parents allows to determine with sufficient reliability the patient's behavior. An eventual worsening of the hump requires further investigation on the behavior of the patient.

Compliance to treatment is considered optimal when the difference between the hours of bracing prescribed and those spent wearing the brace is smaller than 2. The compliance was reassessed through questionnaires mailed to 67 patients approximately 1 year after the end of weaning. Responses were received from 62 patients (93%). A complete compliance to treatment was declared by 54 patients (88%). The high degree of acceptance of the PASB brace derives from its characteristics:

-is less burdensome than other commonly used braces

-is perfectly hidden under clothes

-allows considerable freedom of movement of the trunk.

All this has a significant impact on the patient's quality of life (QoL). Indeed, patients treated with the PASB had higher QoL scores at the Brace Questionnaire (BrQ) compared with those treated with the Lyon brace [27].

## Discussion

The conservative treatment of adolescent idiopathic scoliosis is traditionally pursued through the use of braces whose mechanical action is expressed by the three-point principle. The biomechanical rationale underlying orthoses currently used in clinical practice focuses on the exploitation of external forces by means of pads applied to the brace. This biomechanical approach allows to halt the curve progression, but hardly obtains a significant and stable correction of the deformity. In particular, the scientific literature shows that current orthoses rarely induce the derotation of vertebral segments included within thoraco-lumbar and lumbar curves. Since the intervertebral rotation and the relative twisting of the motion segments represent both the anatomopathological features of the deformity and the main biomechanical factor responsible for the curve progression, the failure to achieve a derotation explains the limited success obtained by orthoses currently used in clinical practice.

In an effort to improve the efficacy of bracing, considerable research has been directed toward the development of orthoses characterized by a new geometry. This aim has been pursued without substantial innovation of the biomechanical principles. This has generated skepticism by some authors about the possibility of actively interfering on the progression of scoliosis via nonoperative treatment.

A better understanding of the elastic behavior of the intact spine and the analysis of the biomechanical changes of a scoliotic spine have provided a new impetus to the conservative treatment of idiopathic scoliosis [28,29]. In this context, the PASB allows an original biomechanical approach. In fact, through its peculiar geometry, the PASB is able to constrain the trunk movements, exploiting the internal forces generated to induce a reversal of stress loads acting on the scoliotic spine. This promotes the recovery of vertebral symmetry. Particularly significant in this regard is the derotation of the apical vertebra of the curve, which maintains stable over time. Hence, the prospect of conservative treatment is not longer limited to halting the curve progression, but extends to the achievement of its recovery.

The analysis of outcomes obtained with the PASB, confirm our biomechanical premises, leading to a first, albeit partial, identification of factors that influence the response of the scoliotic spine to the PASB action. These are represented by the modification of the biomechanical properties of visco-elastic structures in relation to growth and the entity of deformation.

A better definition of the biomechanical parameters identified, such as the extent of the spinal twist and the variation of the G modulus (modulus of rigidity to torsion) in function of the discal deformation and the patien's age, together with the identification of other possible contributing factors, represent, in our opinion, the requisites to pursue the optimization of conservative treatment of idiopathic thoraco-lumbar and lumbar scoliosis.

## Conclusions

Our results confirm the validity of a different biomechanical approach for nonoperative treatment of scoliosis. The efficacy of the PASB derives not only from its unique biomechanical features, but also from the simplicity of its design, construction and management. The different modalities for its construction and the thrust application described for other types of braces are not possible for the PASB brace.

## Abbreviations

BrQ: Brace Questionnaire; PASB: Progressive Action Short Brace; QoL: quality of life; SIAS: Spina iliaca anterior superior; TLSO: Thoraco-lumbar-sacral orthosis.

## Competing interests

The authors declare that they have no competing interests.

## Authors' contributions

All authors contributed equally to this work. All authors read and approved the final manuscript.

## References

[B1] Lupparelli S Pola E Pitta L Mazza O De Santis V Aulisa L Biomechanical factors affecting progression of structural scoliotic curves of the spine Stud Health Technol Inform 2002 91 81 85 15457699

[B2] Lupparelli S Tamburrelli F Padua R Marrocco R Aulisa L The progressive action short brace (PASB): A Different approach to the conservative treatment of thoracolumbar and lumbar idiopathic curves 1999 Research into Spinal Deformities 2, IOS Press 281 284

[B3] Aulisa L Vinciguerra A Tamburrelli F Lupparelli S Di Legge V Biomechanical Analysis of the Elastic Behaviour of the Spine with Aging 1997 Research into Spinal Deformities l, IOS Press 229 231

[B4] Castro FP Jr Adolescent idiopathic scoliosis, bracing, and the Hueter-Volkmann principle Spine J 2003 3 180 185 10.1016/S1529-9430(02)00557-0 14589197

[B5] Weiss HR Hawes MC Adolescent idiopathic scoliosis, bracing and the Hueter-Volkmann principle Spine J 2004 4 484 485 15246310 10.1016/j.spinee.2004.01.015

[B6] Stokes IA Mechanical modulation of spinal growth and progression of adolescent scoliosis Stud Health Technol Inform 2008 135 75 83 18401082

[B7] Stokes IA Burwell RG Dangerfield PH Biomechanical spinal growth modulation and progressive adolescent scoliosis - a test of the 'vicious cycle' pathogenetic hypothesis: Summary of an electronic focus group debate of the IBSE Scoliosis 2006 1 16 10.1186/1748-7161-1-16 17049077 PMC1626075

[B8] Grivas TB Vasiliadis E Malakasis M Mouzakis V Segos D Intervertebral disc biomechanics in the pathogenesis of idiopathic scoliosis Stud Health Technol Inform 2006 123 80 83 17108407

[B9] Di Benedetto A Vinciguerra A Pennestrì E Aulisa L Biomechanics of scoliosis using a new type of brace Ediz 1981 Roma: ESA 1 31

[B10] Stokes IAF Hueter-Volkmann effect. State of the Art Reviews Spine 2000 14 349 357

[B11] Quagliarella L Aulisa L Lupparelli S Tartarone M Pressures exerted by braces used for conservative treatment of idiopathic scoliosis: an experimental measurement Health Technol and Inform 1997 37 255 258

[B12] Rigo M Negrini S Weiss H Grivas T Maruyama T Kotwicki T SOSORT consensus paper on brace action: TLSO biomechanics of correction (investigating the rationale for force vector selection) Scoliosis 2006 1 11 10.1186/1748-7161-1-11 16857045 PMC1553475

[B13] Di Benedetto A Vinciguerra A Pennestrì E Aulisa L Biomechanics of Scoliosis Using a New Type of Brace Proceedings of the 8th Canadian Congress of Applied Mechanics 1981 Moncton, .N-.B, Canada 785 786

[B14] Aulisa L Tranquilli Leali P Valassina A Merolli A Jane Dansereau Treatment of lumbar and thoraco-lumbar curves by the corrective and derotating action of a short brace of new design International Symposium on 3-D Scoliotic Deformities 1992 New York: Gustav Fischer Verlag 317 324

[B15] Beausejour M Petit Y Grimard G Aubin CE Dansereau J Labelle H Relationships between strap tension, interface pressures and spine correction in brace treatment of scoliosis Stud Health Technol Inform 2002 88 207 211 15456033

[B16] Aulisa L Di Benedetto A Vinciguerra A [Un'analisi biomeccanica del sistema tutore-rachide nelle scoliosi idiopatiche]. (Article in Italian) Arch Putti 1981 31 185 194 7345998

[B17] Moe JH Kettleson DN Idiopathic scoliosis: analysis of curve patterns and the preliminary results of Mil-Waukee brace treatment in one hundred sixty-nine patients J Bone Joint Surg Am 1970 52 1509 1533 5483076

[B18] Bunnell WP Treatment of idiopathic scoliosis Orthop Clin North Am 1979 10 813 827 523083

[B19] Keiser RP Shufflebarger HL The Milwaukee brace in idiopathic scoliosis: evaluation of 123 completed cases Clin Orthop 1976 118 19 24 954276

[B20] Bassett GS Bunnell WP MacEwen GD Treatment of idiopathic scoliosis with the Wilmington brace: results in patients with a twenty to thirty-nine-degree curve J Bone Joint Surg Am 1986 68 602 605 3957986

[B21] Winter RB Lonstein JE, Bradford DS, Winter RB, Ogilvie JW Classification and terminology Moe's Textbook of Scoliosis and Other Spinal Deformities 1995 39 3 Philadelphia: WB Saunders Company 44

[B22] Bunnell WP Treatment of idiopathic scoliosis Orthop Clin North Am 1979 10 813 827 523083

[B23] Aulisa AG Guzzanti V Marzetti E Menghi A Giordano M Aulisa L Correlation between hump dimensions and severity curves in idiopathic scoliosis before and after conservative treatment Spine (Phila Pa 1976) 2011 in press 10.1097/BRS.0b013e3181ee77f9 21224763

[B24] Aulisa L Tamburrelli F Lupparelli S Serra F Pitta L IAF StoKes Treatment of thoracolumbar and lumbar idiopathic scoliotic curves with the progressive action short brace (P.A.S.B.): Analysis of results Research into Spinal Deformities 2 1999 IOS Press 358 361

[B25] Aulisa AG Guzzanti V Galli M Perisano C Falciglia F Aulisa L Treatment of thoraco-lumbar curves in adolescent females affected by idiopathic scoliosis with a progressive action short brace (PASB): assessment of results according to the SRS committee on bracing and nonoperative management standardization criteria Scoliosis 2009 4 21 10.1186/1748-7161-4-21 19765288 PMC2754424

[B26] Aulisa L Lupparelli S Pola E Aulisa AG Mastantuoni G Pitta L Grivas Biomechanics of the conservative treatment in idiopathic scoliotic curves in surgical "grey-area" Healt technology and informatics, 91 V, Research into Spinal Deformities 4, B 2002 IOS Press 412 418 15457767

[B27] Vinciguerra A Aulisa L Quagliarella L Biomechanical instability of the spine in adult scoliosis Prog in Spin Path 1990 5 37 48

[B28] Aulisa AG Guzzanti V Perisano C Marzetti E Specchia A Galli M Giordano M Aulisa L Determination of quality of life in adolescents with idiopathic scoliosis subjected to conservative treatment Scoliosis 2010 5 21 10.1186/1748-7161-5-21 20920196 PMC2958155

[B29] Vinciguerra A Di Benedetto A Aulisa L Sulla determinazione delle caratteristiche elastiche del rachide toraco-lombare. Article in Italian Min Orthop 1984 35 133 138

